# The influence of a full-time, immersive simulation-based clinical placement on physiotherapy student confidence during the transition to clinical practice

**DOI:** 10.1186/s41077-018-0062-9

**Published:** 2018-02-20

**Authors:** Anthony Wright, Penny Moss, Diane M. Dennis, Megan Harrold, Simone Levy, Anne L. Furness, Alan Reubenson

**Affiliations:** School of Physiotherapy and Exercise Science, Curtin University, GPO Box U1987, Perth, WA 6845 Australia

## Abstract

**Background:**

Novice students may have limited learning opportunities during their early exposure to complex clinical environments, due to the priorities of patient care. Immersive, high-fidelity simulation provides an opportunity for physiotherapy students to be exposed to relatively complex scenarios in a safe learning environment before transitioning to the clinical setting. The present study evaluated the influence of immersive simulation on student confidence and competence.

**Methods:**

Sixty penultimate year physiotherapy students completed an 18-day full-time immersive simulation placement. The placement involved students spending 6 days working in each of three core practice areas (cardiopulmonary, musculoskeletal, neurological) in which they interacted with simulated patients portrayed by professional role-play actors. The patient scenarios were developed by groups of expert practitioners and incorporated full documentary and imaging information. Students completed a questionnaire to evaluate their confidence in the clinical environment at the start and completion of each 6-day rotation. Their clinical competence was evaluated at the end of each 6-day rotation using the Assessment of Physiotherapy Practice (APP) tool. In a secondary analysis, the clinical competence of this cohort was evaluated in comparison to a matched cohort of students from the same year group that had not completed an immersive simulation placement.

**Results:**

Student confidence improved significantly in each 6-day rotation (*p* < 0.001); however, it reduced again at the commencement of the next rotation, and there was no cumulative improvement in confidence over the 18-day placement (*p* = 0.22). Students who had completed the immersive simulation placement achieved higher APP (*p* < 0.001) scores in an evaluation of their competence to practice during their subsequent clinical placement.

**Conclusion:**

Immersive simulation provides a beneficial learning environment to enable physiotherapy students to transition from university-based education to working in the clinical environment.

**Electronic supplementary material:**

The online version of this article (10.1186/s41077-018-0062-9) contains supplementary material, which is available to authorized users.

## Background

Real-world clinical training in healthcare rightly places the patient at the center of care such that the focus on student learning may be secondary to the priorities of patient care [1]. Although this is appropriate in terms of patient care, the extent to which students are able to engage in relevant and worthwhile learning experiences within the clinical environment may be limited [2]. A wide spectrum of simulation-based learning activities may be utilized in the training of healthcare professionals [3]. At Curtin University, discrete simulation-based learning activities dispersed throughout the physiotherapy curriculum have demonstrated positive student outcomes [4–6]. Immersive simulation, which fully reflects the interaction between therapist and patient in the clinical environment, provides a valuable learning environment in which students can engage in learning experiences that effectively prepare them for the transition to working and learning in the clinical setting [3, 7].

The modality of immersive simulation has been embraced in physiotherapy and nursing education [8–10]. The advantages of the approach relate to the potential for added clinical and educational value in a teaching environment that is student rather than teacher or patient-centered [11, 12]. A fully simulated clinical placement of several-weeks duration may be designed to include a number of practice areas and a range of cases, which can be presented in a structured manner [13]. This facilitates guaranteed exposure to a particular caseload, with manipulation of the degree of difficulty where appropriate. Patient presentations are standardized, and time may be managed to enhance learning with strategies such as time-outs to pause and discuss [14] or rewinds and replays to facilitate practice in a way that augments experiential learning. Simulation-based clinical practice may also incorporate features to heighten student safety, including the capacity for guided and targeted debriefing [15, 16]. In addition to building clinical competence, this approach may assist in making students more confident in the clinical learning environment.

A recent systematic review [7] has shown that immersive simulation is an effective educational medium to support the development of clinical competence in physiotherapy students. Four large multicenter, randomized controlled trials conducted in Australia evaluated the effect of replacing up to 25% of physiotherapy clinical placement time with immersive simulation [8, 10]. These trials demonstrated that compared to students undergoing traditional placements with full-time interaction with patients, comparable levels of clinical competence were achieved in students completing placements in which 25% of placement time was replaced with immersive simulation. A similar, large, multicenter, randomized controlled trial of nursing education in the USA demonstrated that up to 50% of clinical education time could be replaced with immersive simulation and that students undertaking immersive simulation achieved equivalent educational and competency outcomes to students completing their education entirely in the clinical setting [9].

Blackford and colleagues have shown that a 1-week period of simulation-based training in cardiopulmonary physiotherapy is effective in building student confidence before continuing with a placement in the clinical setting [17]. It might be expected that improvements in student confidence could be linked to improvements in competence, although other researchers have cautioned about the lack of a direct relationship between confidence and competence [18]. It may be that one of the key benefits of immersive simulation is to build confidence and self-belief in novice students prior to entering the clinical environment.

From 2013 to 2015, a consortium of 16 physiotherapy schools in Australia [19] completed a major project, funded by Health Workforce Australia (HWA), to implement immersive high-fidelity, role-play simulation as a component of clinical training in their entry-level education programs (Physiotherapy National Simulation Project) [20]. Three models were developed to facilitate the integration of immersive simulation into clinical placements at different universities across the country. One of the models involved the replacement of the students’ first formal clinical placement with one where 100% of the placement time was committed to immersive simulation. This was termed an introductory placement, and it involved students spending 5–6 days engaged in immersive simulation in each of three main practice areas (cardiopulmonary, musculoskeletal, or neurological) with some additional learning activities to assist students in making the transition to the clinical setting. This model provided the basis for the placement evaluated in this study.

At Curtin University, this introductory, simulation-based placement was implemented with a subset of 60 students in their penultimate year of study. The primary aim of this study was to evaluate changes in the confidence of these students over the course of the placement and in particular to determine if students’ confidence improved cumulatively as they progressed through the placement. As a secondary analysis, the clinical competence of students at the end of their first core clinical placement (cardiopulmonary, musculoskeletal, or neurological) of the final year was compared with that of students who had completed a traditional penultimate year introductory placement that did not involve simulation.

The study sought to address the following key questions:

1. What is the impact of an 18-day simulation-based clinical placement on the self-reported clinical confidence of penultimate year physiotherapy students?
2. Does student clinical confidence improve sequentially as students rotate through three different practice areas during the 18-day placement?
3. During a simulation-based clinical placement, is there an association between self-reported student confidence and student competence as assessed by clinical supervisors?
4. Is there a difference in clinical competence at the end of the first clinical placement completed in a final year, between students who completed simulation-based introductory placement and those who completed a traditional introductory placement in their penultimate year of study?

## Methods

### Participants

Participants were 60 penultimate year Curtin University pre-registration physiotherapy students who were allocated to undertake an 18-day immersive simulation-based introductory placement as a subset of the national physiotherapy national simulation project [20]. Student allocations were made by independent clinical placement administration staff from an overall cohort of 157 students using standard allocation practices, based on numbers and locations, not on student preferences for a simulation or non-simulation-based placement. Of the 60 students who participated in the simulation-based placement, 57 proceeded to undertake a final year clinical placement in the subsequent year.

Ethical approval was provided by the Curtin University Human Research Ethics Committee (HREC) (NEAF HR07/2014) as part of the larger national HWA-funded project. Written informed consent for collection of confidence questionnaire data was provided by students enrolled in the simulation unit. As approved by the Curtin HREC, all APP data from clinical placements were provided to the investigators in de-identified form.

#### Intervention

All participants completed an 18-day simulation-based placement at the end of their penultimate year. The placement was immersive in the sense that students were fully engaged with their simulated patients and supervisors in all aspects of clinical interaction and patient management. The interactions did not focus on a particular component of assessment or treatment or a particular procedural activity. The format of the placement included 18 days of immersive simulation in which students worked with simulated patients. In addition, there was an introductory process at the start of each 6-day period. Table 1 illustrates the similarities and differences between the simulation-based placement and a traditional introductory placement.

**Table 1:** Similarities and differences between traditional introductory placements and the simulation-based introductory placement

	Traditional placement	Simulation-based placement
Time frame	18 days, 7.5 h/day	18 days, 7.5 h/day
Supervision model	Variable—1:2 to 1:6	1:4
Total number of cases seen	Variable	25 (8 or 9 per core area); all students see the same cases
Practice area	One area, variable	3 areas (cardiopulmonary, musculoskeletal, neurological) for all students
Acuity of cases seen	Variable, depending on placement setting and area of practice; unpredictable within each placement	Planned according to stage of learning
Pathologies seen	Variable according to placement; unpredictable within each placement	Planned according to stage of learning
Patient feedback	Variable—none specifically sought	Planned so that all students received feedback on professionalism and communication from professional actors

**Fig. 1:**
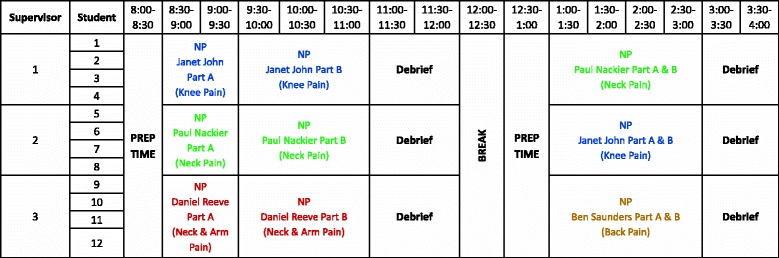
Example of day 2 timetable for a musculoskeletal simulation rotation

Students experienced a planned series of high-fidelity simulated clinical scenarios across the 18 days of the placement, rotating between 6-day blocks allocated to a single core practice area (cardiopulmonary, musculoskeletal, and neurological). All cases were portrayed by simulated patients [21] all of whom were professional role-play actors, and the setting, equipment, and resources such as medical notes, X-Rays, MRIs, and blood tests were prepared to look as authentic as possible. The equipment, environment, and interactions with supervisors and other staff were designed to reflect the clinical environment as accurately as possible. For example, the musculoskeletal block was conducted in the University Physiotherapy Clinic. Simulated patients presented in the waiting area and interacted with the clinic receptionist who then advised the student that their patient had arrived and was ready to commence treatment. In order to facilitate authenticity, before each scenario, students were only provided with the referral information that was likely to be available to them in the clinical setting. In total, all students experienced 25 different scenarios, 8 in musculoskeletal and neurological and 9 in the cardiopulmonary area. The placement lasted for 18 days, divided into three 6-day blocks in which students focused on a specific practice area. Students started each 6-day block working in groups of four, then pairs, and working individually with simulated patients in the final 2 days. Figure 1 shows an example of a timetable for day 2 of the musculoskeletal block for a subgroup of 12 students working in groups of 4. Students participated in supervisor-facilitated debriefing sessions in which they discussed the patient scenarios that they had all observed. Supervisors were all experienced clinicians. Some were hospital staff who were seconded for the placement and others were university staff who were regularly involved in supervising student clinical placements. Due to the lack of additional clinical responsibilities, supervisors were able to focus entirely on student learning.

All simulation scenarios were specifically written for the physiotherapy national simulation project [19] by teams of expert clinicians and benchmarked across Australia as part of the HWA-funded project, with small modifications made for the local setting in Western Australia. The bank of scenarios was designed to ensure that all core competencies and key pathologies were experienced by students within a single simulation-based placement. Cases were presented in acute care, rehabilitation, and community settings and involved both initial and follow-up treatment sessions [19]. Scenario resources included scripts for actors, photographs of the clinical presentation and set-up, clinical equipment, audio files for auscultation where appropriate, medical and surgical notes, referral letters, imaging (X-Rays, MRIs, CT scans), bed charts, blood tests, as appropriate to the case example. Where needed, additional moulage, dressings, tubing for drips, and drains were also provided to enhance authenticity.

All actors and simulation supervisors underwent at least 5 h of individualized training before the placement. Actors were provided with generic simulation technique training using online NHET-Sim training modules [22], with all actors completing at least the initial core module which provides an overview of simulation education. Actors were also specifically trained to give feedback to students regarding their professional and communication skills and in the more specific details of the subjective and physical presentation of their assigned patient. Actors were provided with a detailed script and photographs or short video clips for their scenario at least 1 week before the start of the placement. Actors then attended a 3-h face-to-face training session approximately 3 days before the start of the placement, run by a coordinating project research officer and clinical supervisors with expertise in each practice area. Simulation supervisor training involved initial completion of a minimum of the two core online NHET-Sim training modules. Staff were then provided with two half-day face-to-face NHET-Sim sessions [22] at which they were trained in the use of specific simulation techniques to be used to improve the learning experience for students. This included manipulation of time through time-outs, rewind, and replay [23]. This allowed a student or supervisor to stop the scenario, discuss any issues or uncertainties, and then rewind and replay the same part of the session, applying their new approach immediately. Supervisors were also trained to facilitate debrief sessions, to address student concerns and uncertainties as well as focusing on the learning objectives of the placement. Supervisors were provided with additional written information in a detailed training manual, developed as part of the resources for the physiotherapy national simulation project.

### Outcome measures

Student confidence was evaluated using the clinical confidence measure utilized in previous physiotherapy simulation studies [8, 10], as shown in Additional file 1: Appendix 1. The questionnaire was modified by the addition of one question and was delivered via an iPad application. The questionnaire was completed by students on day 1 and day 6 for each practice area rotation. This enabled evaluation of changes in confidence for each rotation as well as from baseline to the end of the entire placement. Scores for the 14 questions were grouped into four skills areas; communication (questions 1–4), assessment (questions 5–8), treatment (questions 9–11), and hazard awareness (questions 12–14). This student clinical confidence questionnaire has shown good test-retest intra-rater reliability in a sample of 330 physiotherapy students over 3 weeks, with ICC 0.830 (0.795–0.851) (unpublished data).

Students’ clinical competence was evaluated using the Assessment of Physiotherapy Practice (APP) tool [24, 25]. This is a standardized, valid, and reliable clinical assessment tool used across all physiotherapy schools in Australia and New Zealand, which was developed based on the Australian physiotherapy standards. It contains 20 items across seven domains, with each item scored from 0 to 4 (whereby 0 or 1 = not yet adequately competent; 2 = entry-level standard; 3 = demonstrates comfort; 4 = demonstrates sophistication). Any item can also be scored as “not applicable” if appropriate. These items can be grouped into two sub-scores of professional competence items and clinical competence items [17]. Professional competence items include teamwork and communications skills while clinical competence items include assessment, treatment, and goal setting. For each student, a mean overall grade was calculated, together with mean professional and mean clinical competence grades. APP evaluations were completed by the simulation supervisor at the end of each practice area rotation during the simulation-based placement using an iPad application.

Students completing the simulation-based placement were asked to complete the clinical confidence questionnaire at the start of each 6-day period and again at the end of each 6-day period. Their clinical competence in each practice area was evaluated by their simulation supervisor at the end of each 6-day period.

The APP grade recorded by the clinical placement supervisors at the end of their first final year clinical placement using a standard paper format was collected for those students who had completed the simulation placement and proceeded to the final year of their course. Two students did not progress to complete clinical placements in the subsequent year, and one student withdrew from the course entirely. In addition, APP grades for 57 students from the same cohort who had not completed a simulation-based placement were collected. These students were matched to simulation placement students by gender, course (undergraduate or graduate entry), and the core practice area (cardiopulmonary, musculoskeletal, or neurological) of their first final year placement. De-identified student data was compiled by the clinical placement administration staff, and students were matched sequentially by the research officer.

### Data analysis

SPSS version 22 (IBM) was used to analyze data, with alpha set at *p* < 0.05. All data were found to be normally distributed. Difference in confidence scores from day 1 to day 6 in each core practice area, as well as change in confidence score for each 6-day period during the placement and from day 1 to day 18 of the placement, were analyzed using paired *t* tests. Confidence scores at the end of each of the 6-day blocks were also analyzed using repeated measures ANOVA. Associations between confidence scores at the end of each 6-day period and APP scores in each practice area were evaluated using Pearson’s correlation coefficient.

In a secondary analysis APP data from the first core area, clinical placement in final year was compared between 57 students who had been allocated to the simulation-based introductory placement and 57 students who had been allocated to a traditional non-simulated introductory placement. Mean total APP grade and mean grades for the professional and clinical competence items were calculated. Group differences in total and sub-score (professional skills, clinical skills) grades were analyzed using independent *t* tests.

## Results

Eighteen male and 42 female students (mean age 22.3 years) participated in the study. Of those students, 45 were enrolled in the Bachelor of Physiotherapy course and 15 in the graduate-entry Master of Physiotherapy course. All participants completed confidence questionnaires at the assigned times except for one participant who did not complete the questionnaire during the third 6-day period in which they were working in the neurology practice area.

### Student confidence scores

There was a clear improvement (*p* < 0.001) in student self-reported confidence following 6 days of immersive simulation in each of the core practice areas (Table 2). The magnitude of improvement was comparable between areas, with day 6 total scores increasing from day 1 by 35.8% for cardiorespiratory, 35.7% for neurology, and 36.0% for musculoskeletal rotations. Confidence improved across all skill areas although students had higher confidence scores for communication and assessment skills than for treatment skills and hazard awareness. This was the case both at the start and the end of the 6-day period (Table 2).

**Table 2:** Change in confidence scores for each practice area during the 18-day placement

	Pre	Post	*t* value	*p* value (2-tailed)
Cardiopulmonary				
Communication score	9.10	16.25	− 24.25	0.001
Assessment score	9.00	16.25	− 23.77	0.001
Treatment score	6.87	11.85	− 23.22	0.001
Hazard awareness score	6.82	12.55	− 20.77	0.001
Total score	31.82	56.90	− 27.91	0.001
Neurology				
Communication score	8.85	16.10	− 23.15	0.001
Assessment score	8.90	15.87	− 21.67	0.001
Treatment score	7.00	11.72	− 20.46	0.001
Hazard awareness score	6.60	12.67	− 20.06	0.001
Total score	31.35	56.35	− 27.58	0.001
Musculoskeletal				
Communication score	8.98	16.27	− 27.29	0.001
Assessment score	8.97	16.24	− 28.70	0.001
Treatment score	7.00	11.83	− 23.20	0.001
Hazard awareness score	6.66	12.44	− 19.01	0.001
Total score	31.61	56.78	− 32.27	0.001

**Fig. 2:**
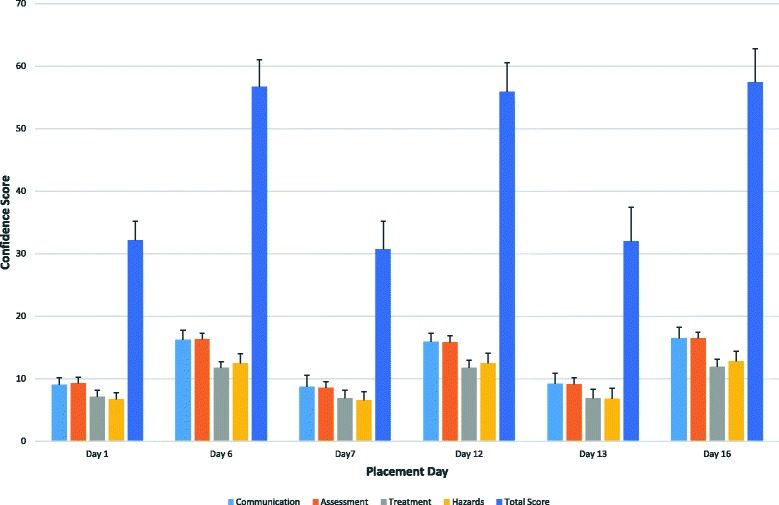
Change in student self-reported confidence scores over the duration of the placement

Although confidence improved from the start to the end of each 6-day period of the placement (Table 3), there was no cumulative improvement over the entire placement (Fig. 2). Instead, at the start of each new block when students transitioned to a new practice area, their confidence scores returned to baseline levels. This pattern was consistent for all sub-categories of the questionnaire. There was no significant difference in total confidence scores at the end of each 6-day block (*F*_2, 176_ = 1.56, *p* = 0.22).

**Table 3:** Change in confidence scores for each 6-day period during the 18-day placement

	Start	End	*t* value	*p* value (2-tailed)
Days 1–6				
Communication score	9.05	16.23	− 29.56	0.001
Assessment score	9.25	16.28	− 23.92	0.001
Treatment score	7.10	11.75	− 22.61	0.001
Hazard awareness score	6.73	12.45	− 21.86	0.001
Total score	32.13	56.72	− 33.48	0.001
Days 7–12				
Communication score	8.73	15.93	− 24.67	0.001
Assessment score	8.52	15.82	− 28.12	0.001
Treatment score	6.88	11.73	− 22.29	0.001
Hazard awareness score	6.55	12.42	− 19.25	0.001
Total score	30.68	55.90	− 30.83	0.001
Days 13–18				
Communication score	9.15	16.46	− 21.68	0.001
Assessment score	9.10	16.25	− 21.70	0.001
Treatment score	6.88	11.91	− 21.95	0.001
Hazard awareness score	6.80	12.80	− 19.03	0.001
Total score	31.97	57.42	− 25.00	0.001

### Correlation of confidence and APP scores

There were significant positive correlations between total confidence scores at the end of each 6-day period (Table 4). However, there was no correlation between total confidence score and APP score for any of the 6-day periods (Table 5).

**Table 4:** Correlations between confidence ratings at the end of a 6-day period in each core practice area

		Confidence period 1	Confidence period 2	Confidence period 3
Confidence period 1	Pearson correlation	1	0.580	0.598
	Sig. (2-tailed)		0.001	0.001
	*N*	60	60	60
Confidence period 2	Pearson correlation		1	0.701
	Sig. (2-tailed)			0.001
	*N*		60	59
Confidence period 3	Pearson correlation			1
	Sig. (2-tailed)			
	*N*			59

**Table 5:** Correlations between confidence ratings at the end of each 6-day period and APP scores in each of the core practice areas

		APP period 1	APP period 2	APP period 3
Confidence period 1	Pearson correlation	0.150	− 0.055	− 0.004
	Sig. (2-tailed)	0.252	0.676	0.974
	*N*	60	60	59
Confidence period 2	Pearson correlation		0.072	0.091
	Sig. (2-tailed)		0.587	0.494
	*N*		60	59
Confidence period 3	Pearson correlation			0.040
	Sig. (2-tailed)			0.761
	*N*			59

### Student competence grades

In a secondary analysis, the APP grades achieved by students at the end of their first final year core practice area placement were compared between simulation students and those who had completed a traditional introductory penultimate year placement. Students who completed the simulation-based placement achieved significantly higher APP grades overall and significantly higher grades in the professional and clinical sub-categories of the APP (*p* < 0.001 for all) (Table 6).

**Table 6:** Comparison of APP scores between simulation and non-simulation students

APP grades	Simulation/non-simulation	*N*	Mean	SD	
Overall score	Simulation students	57	3.05	0.512	*t* = 5.450 *p* < 0.001
	Non-simulation students	57	2.55	0.466	
Professional skills	Simulation students	57	3.18	0.521	*t* = 5.086 *p* < 0.001
	Non-simulation students	57	2.69	0.522	
Clinical skills	Simulation students	57	2.89	0.549	*t* = 4.730 *p* < 0.001
	Non-simulation student	57	2.44	0.476	

## Discussion

The findings from this study show that an introductory simulation-based placement significantly improves student confidence in their ability to undertake clinical practice in each of the core practice areas. Students who completed the simulation-based placement also showed increased clinical competence in a subsequent clinical placement compared to those who had not.

An important aspect of an introductory simulation-based placement may be allowing novice students to become comfortable in the clinical environment in a very low-risk situation and enabling them to become familiar with the learning process as it develops within the clinical setting. Improvement in student confidence following a simulation-based placement has been demonstrated by Blackford and colleagues [17] in a model that involved replacing the first week of a 5-week clinical placement with immersive simulation. The current study demonstrated a similar improvement in student confidence over a 6-day period of simulation-based training.

However, the main finding from the current study was that there was no cumulative improvement in student confidence over the course of the entire placement period. Student confidence improved over the 6-day period in which they worked in one core practice area, but when students then switched to another practice area, their confidence levels returned to baseline (Fig. 2). Improvements in confidence from the beginning to the end of the placement were no greater than the improvements that occurred within each 6-day period. This suggests that students’ clinical confidence is linked to area-specific knowledge and skills, which increase across the period spent working in a practice area but does not transfer between areas. Blackstock and colleagues showed a similar improvement in student confidence after 1 week of immersive simulation, but the students’ confidence did not improve further when they then spent an additional 3 weeks working with real patients in the cardiopulmonary practice area [8]. It appears that student confidence increases rapidly during simulation training, but there may then be a limit to further improvement.

The lack of confidence transfer between practice areas may be a reflection of the particular learning experiences of this cohort of students in earlier years of their course. At Curtin University, students complete discrete units of study in the cardiopulmonary, musculoskeletal, and neurological areas, in contrast to other courses where there may be greater integration across core practice areas. Previous anecdotal evidence from Curtin physiotherapy students suggests that they, therefore, tend to view these areas as separate domains, as also emphasized by student feedback at the end of the placement. This perception was unintentionally reinforced by the particular structure of the introductory simulation-based unit; three distinct learning experiences in which students worked with simulated patients focused only on each of the three core practice areas and in which they worked with supervisors who were specialists in each of those areas. In order to build student confidence more progressively over the course of the placement, it might be beneficial to place greater emphasis on some of the more generic skills that students develop while on placement or to structure the placement in a different manner with students moving more fluently between different practice areas throughout the placement.

There was no correlation between confidence scores and APP ratings of student competence in each of the practice area rotations. This finding suggests that the simulation-based placement enhances student confidence irrespective of their overall performance in the clinical environment. In a systematic review, Boling and Hardin-Pierce [18] noted that high-fidelity simulation improved the confidence and knowledge of acute care staff in the intensive care setting, but they also noted that there was no direct relationship between competence and confidence in a number of studies and emphasize the importance of evaluating both constructs, which appears to support our finding.

The placement model evaluated in this study involved students spending 100% of their clinical placement time in a fully immersive simulation-based placement. During the 18-day placement, they only worked with simulated patients. Previous research has demonstrated that simulation can be used to replace 25% and up to 50% of placement time without a negative impact on the development of student competence. In order to evaluate the effect of a 100% simulated placement on student competence, we conducted a secondary analysis in which we compared the APP grades achieved by students who had completed the simulated placement with a group of their matched peers who completed traditional placements. The findings from this analysis indicated that simulation students achieved superior APP scores. This was a positive finding in support of the simulation placement. The finding that students who had completed a simulation-based placement achieved superior APP scores has also been demonstrated previously in the cardiopulmonary area [8].

As this was not a randomized, controlled trial, it is important to acknowledge some limitations of this finding. It is important to consider that it may be a reflection of more time spent working in the particular practice area. Students who completed the simulation-based introductory unit had at least 1-week experience in the practice area prior to starting their final year placement, which was of 5-week duration. For some of the students who completed a traditional introductory placement, the final year placement may have been their first clinical placement in that practice area.

It is recommended that a randomized controlled trial should be conducted in order to fully address the question of whether students completing an entirely simulation-based placement can achieve superior competency outcomes in the clinical setting to students completing a non-simulation-based preparatory placement. It should also be acknowledged that the APP is an evaluation tool predominantly used in Australia. There would be value in further international research using other competency measures to evaluate the outcomes achieved following a fully simulation-based placement.

## Conclusion

This study found that an 18-day simulation-based placement that rotated students through three core practice areas significantly improved students’ confidence in their ability to perform in the clinical setting. The improvement in confidence was very specific to each practice area with no carry-over between practice areas. While students showed significant improvements in confidence during the placement, there was no correlation between confidence and clinical competence demonstrated during the placement. A secondary analysis demonstrated that students who had completed the simulation-based placement demonstrated significantly better clinical competence in a subsequent placement than students who had completed a traditional introductory placement.

## Additional file

Additional file 1: Student confidence questionnaire. (PDF 123 kb)
