# Supplementary material for: The influence of a full-time, immersive simulation-based clinical placement on physiotherapy student confidence during the transition to clinical practice
Source: Adv Simul (Lond). 2018 Feb 20;3:3. doi: 10.1186/s41077-018-0062-9 (PMC5819286; doi:10.1186/s41077-018-0062-9)
Supplement: Supplementary file 1 — Student confidence questionnaire. (PDF 123 kb) [file 41077_2018_62_MOESM1_ESM.pdf]

HealthWorkforce AUSTRALIA

An Australian Government Initiative

This Project was made possible due to funding made available by Health Workforce Australia as an Australian Government Initiative

Student Pre-Simulation Unit Questionnaire

We would like you to evaluate your level of confidence in the following aspects of clinical practice before you start your simulation unit. We will also ask you to complete this after you have finished, to see if there is any change.

Please tick a box for each item that best describes your confidence rating:

1	2	3	4	5	NA
Not at all confident	A little unconfident	Undecided	Fairly confident	Very confident	Not applicable

How confident do you feel about assessing and treating Cardio-respiratory / Neuro / Orthopaedic / Musculoskeletal patients *(please circle the correct patient type for your simulation unit).* ***Please be honest*** (your supervisor will NOT see this).

	1	2	3	4	5	NA
I feel confident that I can:						
1. Communicate effectively with patients verbally						
2. Communicate effectively and professionally with other clinicians (eg writing reports, letters, etc)						
3. Write effective SOAP / medical notes						
4. Provide effective written information for patients						
I feel confident that I can:						
5. Conduct an *efficient* and *thorough* subjective assessment						
6. Conducting an *efficient* and *thorough* physical assessment						
7. Accurately interpret my assessment findings to make a clinical hypothesis						
8. Set appropriate goals in collaboration with the patient						
9. Select appropriate treatment / interventions as a result of my assessment and hypothesis						
I feel confident about:						
10. Performing treatments & interventions						
11. Progressing interventions appropriately for a particular patient						
12. Identifying safety hazards in a particular clinical situation						
13. Responding quickly and appropriately to safety hazards						
14. I feel confident that I am aware of my own limitations						

Thank you for taking time to provide your input.
Enjoy your simulation placement!
